# Neuroantigen-Specific CD4 Cells Expressing Interferon-γ (IFN-γ), Interleukin (IL)-2 and IL-3 in a Mutually Exclusive Manner Prevail in Experimental Allergic Encephalomyelitis (EAE)

**DOI:** 10.3390/cells1030576

**Published:** 2012-08-24

**Authors:** Alexey Y. Karulin, Stefan Quast, Maike D. Hesse, Paul V. Lehmann

**Affiliations:** 1 Vice President R&D, Cellular Technology Ltd., 20521 Chagrin Blvd., Cleveland, OH 44122, USA; 2 Cognitive Neurology Section, Institute of Neuroscience and Medicine (INM-3), Research Center Juelich, Juelich, Germany; Email: sq@immunospot.com (S.Q.); m.hesse@fz-juelich.de (M.D.H.); 3 President and CEO, Cellular Technology Ltd., Cleveland, OH 44122, USA; Email: pvl@immunospot.com

**Keywords:** EAE/MS, Neuroimmunology, Two-color ELISPOT, Dual color ELISPOT Memory T cells, Th1 cells, cytokine co-expression, polyfunctional T cells

## Abstract

Experimental allergic encephalomyelitis (EAE) is mediated by neuroantigen-specific pro-inflammatory T cells of the Th1 and Th17 effector class. Th-17 cells can be clearly defined by expression of IL-17, but not IFN-γ, IL-2 or IL-3. Th1 cells do not express IL-17, but it is unclear presently to what extent they co-express the cytokines canonically assigned to Th1 immunity (*i.e.*, IFN-γ, IL-2 and IL-3) and whether CD4 cells producing these cytokines indeed belong to a single Th1 lineage. It is also unclear to what extent the Th1 response in EAE entails polyfunctional T cells that co-express IFN-γ and IL-2. Therefore, we dissected the Th1 cytokine signature of neuroantigen-specific CD4 cells studying at single cell resolution co-expression of IFN-γ, IL-2 and IL-3 using dual color cytokine ELISPOT analysis. Shortly after immunization, in the draining lymph nodes (dLN), the overall cytokine signature of the neuroantigen-specific CD4 cells was highly type 1-polarized, but IFN-γ, IL-2, and IL-3 were each secreted by different CD4 cells in a mutually exclusive manner. This single cell – single cytokine profile was stable through the course of chronic EAE–polyfunctional CD4 cells co-expressing IL-2 and IFN-γ presented less than 5% of the neuroantigen-specific T cells, even in the inflamed CNS itself. The neuroantigen-specific CD4 cells that expressed IFN-γ, IL-2 and IL-3 in a mutually exclusive manner exhibited similar functional avidities and kinetics of cytokine production, but showed different tissue distributions. These data suggest that Th1 cells do not belong to a single lineage, but different Th1 subpopulations jointly mediate Th1 immunity.

## Abbreviations

ELISPOT: enzyme-linked immunospot
EAE: experimental autoimmune encephalomyelitis
PLP: proteolipid protein
CNS: central nervous system
TCR: T cell receptor
PLP: proteolipid protein
PBST: phosphate buffer saline with tween-20
dLN: draining lymph nodes
HRP: horseradish peroxidase
ALPH: alkaline phosphatase
SFU: spot-forming units
MS: multiple sclerosis
IL: interleukin
Th: T helper
IFN: interferon
IFA: incomplete Freund's adjuvant
TNF: tumor necrosis factor
CNS: central nerve system
APC: antigen presenting cell
CFA: complete Freund's adjuvant
ND: not defined

## 1. Introduction

While considerable progress has been made in understanding the differentiation of CD4 cells into effector cell lineages that express IFN-γ (Th1), IL-4 (Th2) [1] and IL-17 (Th17) [2,3], at present it is unclear to what extent individual T cells within these subsets co-express other cytokines. In the absence of this information, it remains largely unknown how precise and versatile T cells are in implementing the individual effector functions that result from the local production of each of the different cytokines.

Increasing evidence suggests that the expression of the individual cytokines is tightly regulated. Thus, when TCR-transgenic CD4 cells were studied at the single cell level by dual label *in situ* hybridization [4,5] or by two color ELISPOT assays [6], they were found to express either IL-2, IL-3, IL-4, IL-5, or IFN-γ but not to co-express these cytokines. Data obtained by single-cell cytokine RT-PCR showed that *in vivo*-primed TCR transgenic CD8 memory T cells also express IL-2 and IFN-γ in a mutually exclusive manner, but that they can co-express these cytokines after subsequent stimulations with antigen [7].

Chronically restimulated T cells, including T cell lines and clones, are prone to lose differentiation in cell culture, to undergo replicative senescence after about 15 cycles of proliferation *in vitro* [8] and to develop chromosomal aberrations [9], all of which can affect cytokine expression/co-expression. While such senescent cells can be sustained in culture, it is unclear whether they can survive *in vivo*. It is still to be determined to what extent the cytokine expression patterns established for T cell clones, T cell lines and *in vitro*-propagated TCR-transgenic cells reflect T cell biology *in vivo* and whether T cells progress towards cytokine co-expressing phenotypes upon continuous stimulation *in vivo*, for example, when chronically stimulated in an autoimmune setting.

T cells co-expressing two or more Type-1 cytokines including IFN-γ and IL-2 apparently mediate increased protection in anti-viral [10,11,12,13], anti-bacterial [14,15,16], anti-tumor [17] or vaccine-induced [18,19,20,21,22,23] immune responses. It was demonstrated recently, that even such polyfunctional T cells may not actually co-express multiple cytokines at any given time, but switch production from one cytokine to another during the measurement period [2]. In that study, mitogenic polyclonal stimulation of T cells was used due that assay’s limitation to work with low frequency antigen-specific T cells following physiological stimulation by APC. Therefore, even for polyfunctional T cells the cytokine co-expression patterns remain controversial. 

There are several reasons why so little is known about the cytokine signature of CD4 T cells *in vivo*. During infections, antigen-specific CD8 cells can undergo considerable clonal expansions, and can reach high frequencies (> 0.01%) in the blood when they can be reliably studied by flow cytometry. Antigen-specific CD4 cells rarely reach such frequencies in PBMC. Therefore, cytokine signatures of CD4 cells has been primarily studied relying on T cell lines and clones, including all the *in vitro* artifacts associated with long term cell culture. In addition, intracytoplasmic staining (ICS) for cytokines, and its detection via flow cytometry requires cells to be treated with secretion inhibitors and signal enhancers that profoundly interfere with cell biology. ICS protein detection does not account for post-translational regulation, *i.e.*, for the biologically relevant, actual secretory activity. ELISPOT in contrast is suited to measure (a) the actual cytokine secretion by (b) pharmacologically untreated T cells, (c) following physiological stimulation, and most importantly, (d) also for low frequency antigen-specific CD4 cells, directly *ex vivo*, avoiding pitfalls of prolonged *in vitro* tissue culture. Dual color ELISPOT assays have the added advantage that during the entire measurement period, both analytes are captured around the secreting cells. A cell will appear double positive if it produces both analytes, e.g., during a 24 h capture period. Cells will also appear double positive if they initially secrete one analyte, and then switch to the production of the other. The detection of single positive cells, therefore, clearly establishes that that cell did not co-express nor did it switch cytokines during the observation period. For all these reasons, ELISPOT measurements of cytokine expression/co-expression are closer to *in vivo* reality, and might provide novel insights into CD4 cell immunobiology. 

Both Th1 and Th17 cell subsets were shown to be able to mediate autoimmune pathology in experimental allergic encephalomyelitis (EAE) and multiple sclerosis (MS) [24,25,26,27,28,29]. Since it is well established that IL-17 producing CD4 cells do not secrete the Th1 cytokines IFN-γ, IL-2 and IL-3 [30,31] the primary aim of this study was to establish the expression/co-expression of canonical Th1-type cytokines in the autoimmune CD4 cell response against proteolipid protein (PLP) peptide 139-151 in SJL mice [32]. In our previous work, using the same EAE model and dual color IFN-γ/IL-17 ELISPOT assays, we confirmed that these two cytokines are not co-expressed by PLP:139-151-specific CD4 cells at any time of the disease [33]. These findings substantiate that in this EAE model Th17 and Th1 cells are separate lineages. The question remains open whether these autoreactive Th1 cells represent a single lineage. Studying T cell clones, Th1 cells were originally described to co-express IL-2 and IL-3 with IFN-γ [34]. Later it became evident that IL-2 and IFN-γ frequently are not co-expressed, and that a subpopulation of T cells that co-expresses them, so called polyfunctional T cells, play a key role in protective immunity [10,11,12,13,14,15,16,17,18,19,20,21,22,23]. Little is known about polyfunctional CD4 cells in EAE. We sought to establish their frequency early in the autoimmune response, and whether it increases in the course of chronic EAE, as seen after ongoing T cell stimulation *in vitro* (discussed above). We also studied whether the autoimmune CD4 cells in the target organ, the CNS, would differ from those residing in the immune periphery (dLN and spleen) in their cytokine signatures, functional avidities, and the kinetics of cytokine production, and whether these parameters would change over the course of chronic-relapsing immune pathology. Overall, we aimed at defining how precise the autoreactive T cells are in meditating effector functions via their cytokine production, as required for the better understanding of T cell mediated autoimmune pathology and its regulation.

## 2. Results

### 2.1. The Type 1 Cytokine Signature of the PLP: 139-151-Specific CD4 Cell Population; IL-4 Bystander Reaction in Splenic APC

**Figure 1:**
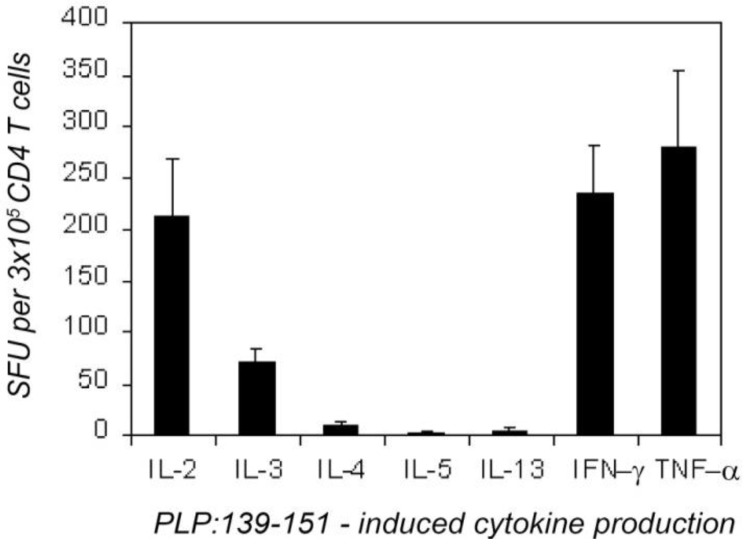
Overall type-1 cytokine profile of proteolipid protein (PLP):139-151-specific CD4 cells 7 days after the immunization with PLP:139-151. CD4 cells (3 × 10^5^/well) purified from draining lymph nodes (dLN) were tested with naive syngeneic (SJL) LN cells (5 × 10^5^/well) in the presence of a maximally stimulatory dose of PLP:139-151 peptide (20 mM). Enzyme-linked immunospots (ELISPOTs) (SFU) were counted by an image analyzer. The results were obtained in four independent experiments, each with four–six mice tested individually. These data are expressed as the mean + SD spots forming cells per 3 × 10^5^ CD4 cells tested.

First, we established the overall cytokine profile of the PLP: 139-151-specific CD4 cells 7 days after immunizing with this peptide. CD4 cells were purified from the dLN of the immunized mice and were tested on naive LN cells functioning as APC. As shown in Figure 1, these CD4 cells produced IFN-γ, IL-2, IL-3 and TNF-α; the frequency of the cells that secreted these cytokines was in the range 50–300 per 3 × 10^5^ CD4 cells plated per well. Confirming our data obtained with other antigen systems [35,36] we found that the number of the nominal antigen (here PLP:139-151)-induced cytokine spots was a linear function of the number of primed CD4 cells plated, and that naive or control immunized mice did not produce these cytokines when challenged with the PLP:139-151 peptide (<1:1,000,000, data not shown). *In vivo*-primed PLP: 139-151-specific CD4 memory cells were detected, therefore in these assays, in the absence of bystander cytokine production by other cells. The PLP:139-151-primed CD4 cells produced IL-5 and IL-13 in frequencies <1:300,000 (Figure 1) and IL-4 producing CD4 cells were in the 10 per 3 × 10^5^ range (1:30,000): that is, the IL-4 producing CD4 cells were outnumbered more than 20 fold by IL-2 and IFN-γ -producing CD4 cells (both of which occurred in frequencies ~1:1,300). Essentially identical results were obtained when unseparated dLN cells were tested, or when purified CD4 cells of PLP: 139-151-immunized mice were studied with naive spleen cells or with thymocytes functioning as APC. It is important to note, however, that the use of LN (or thymic APC) was found to be critical for revealing the cognate IL-4 signature of CD4 cells. When splenic APC were used for the same type of experiments, the induction of vigorous antigen-specific IL-4 production was seen (Table 1). However, this IL-4 was not produced by T cells: it was an IL-3-driven bystander reaction by non-T, non-B cells, most of which were basophils [37].

**Table 1:** PLP:139-151-induced co-expression of IL-2, IL-3, IL-4, and IFN-γ in freshly isolated memory CD4 T cells. SJL mice were immunized with PLP:139-151 peptide and at the time points indicated, CD4 cells were isolated from the dLN or the spleen. CD4 cells were tested in serial dilutions starting at 3 × 10^5^ cells per well with 5 × 10^5^ APC (irradiated naive SJL spleen cells) for the recall response to PLP:139-151 (20 μM) performing the specified double color cytokine ELISA spot assays. Double color ELISPOT assays were of 48 h duration. Data for individual mice representative of groups of 4–24 mice tested per time point are shown. The numbers of single-positive and double-positive (DP) cells were measured by image analysis The % DP was calculated as the percentage of double-cytokine-producing cells from the total number of cells producing both cytokines: % DP = DP × 100%/(DP + Cytokine 1 only + Cytokine 2 only). The mean spot numbers of 3–4 replicate wells are shown for 3 × 10^5^ cells per well with SD < 10%. The standard deviation for all % DP calculated was less than % DP ± 1%.

Time	Disease	Organ	IFN-γ/IL-2 assay				IFN-γ /IL-3 assay				IFN-γ/IL-4 assay			
(days)	state		IFN-γ	IL-2	DP	%DP	IFN-γ	IL-3	DP	%DP	IFN-γ	IL-4	DP	%DP
2	No disease	LN	4	29	3	8.3	5	6	1	8.3	5	9	0	0.0
		Spleen	2	6	0	0.0	4	2	0	0.0	3	47 **	0	0.0
		CNS *	0	0	0	0.0	0	0	0	0.0	0	0	0	0.0
3	No disease	LN	41	104	12	7.6	59	62	8	6.2	51	14	0	0.0
		Spleen	6	23	1	3.3	4	7	0	0.0	9	127 **	0	0.0
		CNS *	0	0	0	0.0	0	0	0	0.0	0	0	0	0.0
7	No disease	LN	224	213	21	4.6	232	62	8	2.6	240	9	1	0.0
		Spleen	356	217	23	3.9	335	86	9	2.1	349	218 **	7	0.0
		CNS *	1	2	0	0.0	3	0	0	0.0	1	0	0	0.0
12	First paralysis	LN	46	183	7	3.0	43	36	5	5.9	52	7	0	0.0
		Spleen	408	210	22	3.4	397	114	13	2.5	429	236 **	4	0.0
		CNS*	932	374	49	3.6	971	372	47	3.4	938	8	1	0.0
21	Second paralysis	LN	45	139	4	2.1	52	53	6	5.4	47	6	0	0.0
		Spleen	116	202	7	2.2	115	71	9	4.6	121	223 **	2	0.0
		CNS *	307	108	11	2.6	288	103	10	2.5	296	14	1	0.0
55	After three relapses	LN	47	76	5	3.9	49	33	2	2.4	41	3	0	0.0
		Spleen	140	201	12	3.4	151	74	11	4.7	148	192 **	2	0.1
		CNS *	119	62	8	4.2	107	42	5	3.2	111	4	0	0.0

* Spinal cord mononuclear cells (frequencies shown per 3 × 10^5^) were tested in the presence of 5 × 10^5^ exogenous splenic APC; ** Bystander IL-4 production by non-T/non-B splenic cells activated in the presence of antigen-stimulated CD4 T cells [37].

This cytokine profile of the CD4 memory cell pool established in single color ELISPOT assays classifies it as perfectly matching the Th1 definition. Therefore, Figure 1 sets the basis for dissecting the Type 1 cytokine profile at the single cell level while studying cytokine co-expression patterns using the dual color ELISPOT methodology. 

These data show that in the inductive phase of the autoimmune response, the peripherally primed PLP: 139-151-specific CD4 memory cell pool is highly type 1 polarized. Does this overall type 1 cytokine signature reflect the secretory activity of classic Th1 cells that at the single cell level co-express these cytokines?

**Figure 2:**
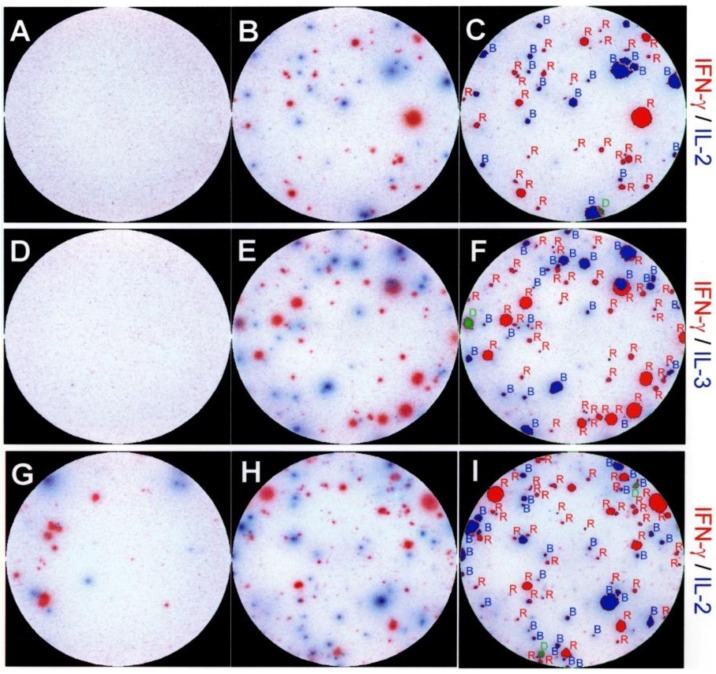
Representative images of double color ELISPOT wells showing dissociated production of interleukin (IL)-2, IL-3 and IFN-γ by individual PLP:139-151 specific CD4 cells. In panels (A-F) CD4 cells purified from dLN seven days after the immunization with PLP:139-151 peptide. In panels (G-I) freshly isolated spinal cord mononuclear cells were tested with naive splenic APC on day 21, during the second episode of paralytic EAE. The medium control wells are shown on the left (A,D,G), wells containing the PLP:139-151 peptide (at 20 mM) are shown in the middle (B,E,H). The cytokine pair measured is specified on the right whereby the color of the substrate and the name of the cytokine are matched. The wells shown have been selected from serial dilutions of T cells; for better visual representation, they were also chosen to have optimal cell density and comparable numbers of red and blue spots. The result of two color image analysis of the peptide-stimulated wells (B,E,H) is shown in the panels on the right (C,F,I) as an overlay of the analyzed image and the original image. Single positive (blue or red spots) are outlined and labeled in red or blue, double positive spots are marked in green. Two color image analysis was performed as described previously [6] and in *Experimental Section 4.3*.

### 2.2. Dissociated Expression of IL-2,-3,-4 and IFN*-γ* in Individual PLP: 139-151-Specific CD4 Cells Prevails in the Inductive Phase of the Autoimmune Response

We performed dual color ELISPOT assays to study the cytokine co-expression by PLP: 139-151-secific CD4 cells. The data obtained for different time points after the immunization, and testing different organs are summarized in Table 1; examples of dual color assay results are shown in Figure 2. In total, we analyzed the cytokine signature of ~200,000 individual peptide-specific CD4 cells 7 days after immunization. The results showed that only 4.6% of these cells co-expressed IFN-γ and IL-2. This number also includes cells that would have switched from the production of one cytokine to the other during the 48 h assay duration, as those too appear double-positive. The vast majority (95.4%) of the peptide-specific CD4 cells were single-positive, producing either IFN-γ or IL-2. 

The frequency of CD4 cells that co-expressed IFN-γ and IL-3 was in the 3% range, while essentially no cells (<0.1%) were detected to co-express IFN-γ and IL-4. The absence of IL-5 producing cells (<1:1,000,000) in the face of the relatively high frequency IFN-γ producing CD4 cells (~1:1,300), and the lack of the co-expression of IL-4 and of IFN-γ argue against the stochastic regulation of these cytokines. The co-expression of IL-2, IL-3, and of IFN-γ in the 2%–5% frequency range argues against both, coordinate expression of the type 1 cytokines in Th1 cells, or their stochastic regulation in CD4 cells 7 days after the immunization.

### 2.3. Segregated Expression of Type-1 Cytokines Occurs as Early as 2 Days after the Immunization

The first cytokine-producing CD4 memory cells became detectable on day 2 after immunization (Table 1). These cells produced either IL-2, IL-3, IFN-γ, IL-4, but no IL-5. The frequency of PLP: 139-151-specific CD4 cells that co-expressed IL-2, IL-3 and IFN-γ was between 8 and 0%. As far as IL-4 and IFN-γ producing cells are concerned, these could be detected initially only in the dLN and both occurred in low frequency (<1:30,000). Subsequently, the frequency of IFN-γ producing cells increased while those producing IL-4 stayed in the same low range. The production of IL-4 and IFN-γ at all time points was fully dissociated at the level of individual T cells. The PLP:139-151 specific T cells did not, therefore, go through an extended Th0 state, but directly acquired the single-cytokine-producing phenotype.

### 2.4. Dissociated Expression of IL-2,-3,-4 and IFN*-γ* in Individual PLP: 139-151-Specific T Cells is Maintained during Relapsing EAE

**Figure 3:**
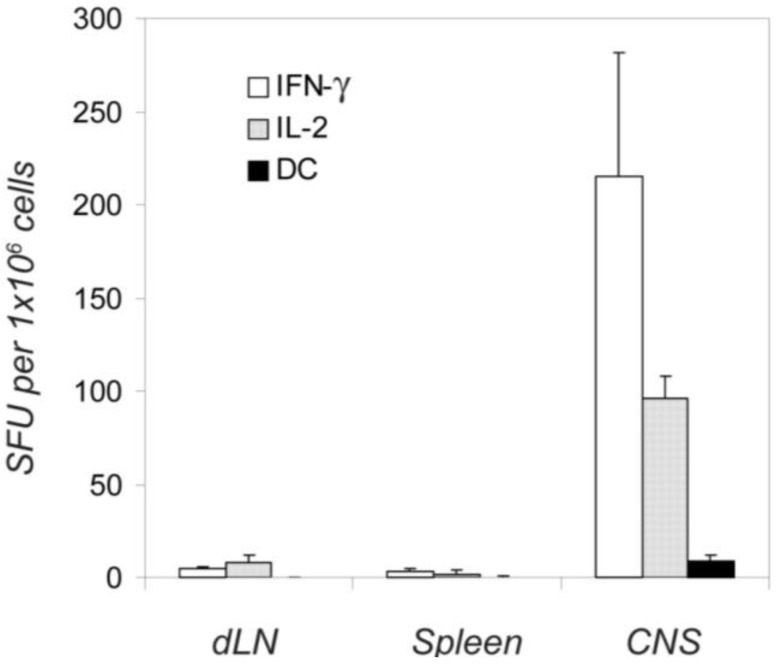
Endogenously activated autoreactive T cells produce either IFN-γ or IL-2, but not both simultaneously (double color, DC). Twelve days after the immunization with the PLP peptide, 1 × 10^6^ unseparated dLN cells, spleen cells, and spinal cord mononuclear cells were plated per well and tested in an IFN-γ /IL-2 double color ELISPOT assay of 48 h duration. Single and double color spots were counted by image analysis. Means and SD of spot forming cells for four independent experiments are shown, with triplicate wells in each experiment.

Immunization of SJL mice with the PLP: 139-151 peptide results in a chronic relapsing form of EAE from which the mice do not recover [32]. We tested mononuclear cells isolated from the spinal cords of such mice. While no PLP:139-151-specific ELISPOT formation was seen in the CNS isolates tested at time points prior to the clinical onset of EAE (days 2, 3 and 7 in Table 1), cytokine-producing cells became detectable at the onset of paralysis. The PLP: 139-151-induced cytokine production by the cells separated from the CNS was blocked by anti-CD4 mAbs, affirming that these cytokines were CD4 T cell derived (data not shown). Unlike the results obtained from the dLN and spleen, where the autoantigen is not presented and where no spontaneous cytokine production was seen in the absence of added peptide, “constitutive” (high background) cytokine production was seen in the cells isolated from the spinal cord (Figure 2 & Figure 3), most likely resulting from the presence of endogenous PLP. These *in situ* activated T cells also showed dissociated production of IFN-γ, IL-2, and IL-3 (Figure 3), and did not secrete IL-4 and IL-5 (data not shown). The addition of PLP: 139-151 induced a ~10 times higher frequency of cognate IFN-γ, IL-2, IL-3, but no IL-4 - (Table 1) and IL-5 - (not shown) - producing cells. When the frequency of cells was studied that co-expressed any combination of these cytokines during the first, second, and third paralysis, it stayed constantly <6%, for the cells isolated from the central nervous system (CNS) itself and for those in the immune periphery alike (Table 1). This outcome was independent of the type of APC used (data not shown, and see below). The data suggest that dissociated cytokine expression does not readily break down and polyfunctional T cells do not arise under these conditions of chronic autoimmune pathology. Comparing the cytokine signatures during phases of remissions and exacerbations of the disease, we did not find qualitative differences in cytokine co-expression (data not shown). 

### 2.5. Dissociated Cytokine Expression in PLP: 139-151-Specific CD4 Cells is Independent of the Antigen Dose

**Figure 4:**
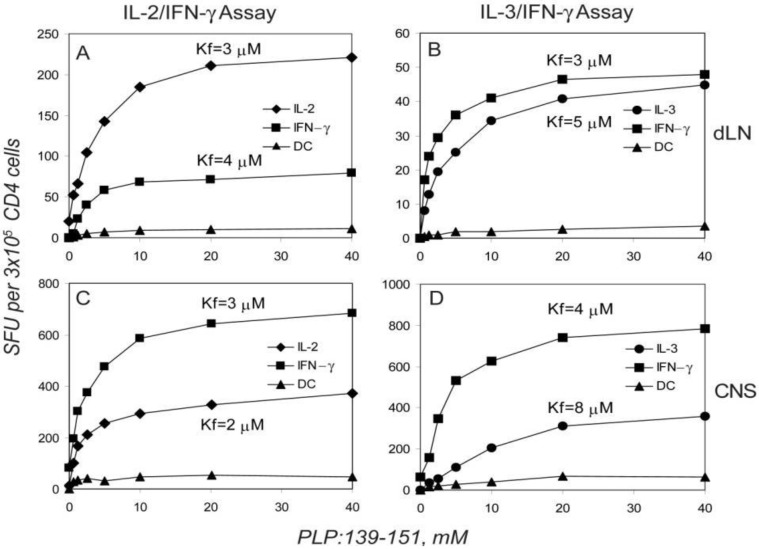
Dissociated production of IL-2, IL-3, and IFN-γ by PLP:139-151-specific CD4 T cells is independent of the peptide dose used for T cell activation; the single cytokine producing CD4 cells have similar functional avidities. Twelve days after immunization, CD4 cells were isolated from dLN (A,B) and mononuclear cells were isolated from the spinal cord (C,D) of SJL mice during the first episode of paralytic encephalomyelitis (EAE). The numbers of single and double-cytokine-producing cells within the 3 × 10^5^ cells plated per well are plotted *vs*. the peptide concentrations used for stimulating the CD4 cells. Isolated CD4 cells were tested with 5 × 10^5^ APC (irradiated naive SJL spleen cells). The peptide concentration leading to the 50% from maximum activation (Kf) defining functional avidity is shown for each single-cytokine dose-response curve (for details see Experimental Procedures). Data shown represent mean values measured from triplicate wells in one of 4 representative experiments. The standard deviations for all data points were less than 5% (comparable in size to symbols used) and are not shown.

Cytokine expression has been linked to the antigen dose, that is, to the signal strength generated by the extent of TCR ligations [38,39,40]. We therefore studied the PLP:139-151 peptide-dose-dependency of cytokine production and co-expression in the dLN, spleen and the CNS. The number of IL-2, IL-3, and IFN-γ spots induced *vs.* the antigen concentration resulted in sigmoidal dose-response curves (Figure 4). While plateau values were reached for all the cytokines tested, the absolute number of spots at maximally stimulatory peptide doses differed for the individual cytokines suggesting different frequencies of cells capable of producing each cytokine. However, the peptide concentrations at which 50% maximal stimulation was reached (Kf value, see *Experimental Section 4.4*), was essentially identical for IL-2, IL-3, and for IFN-γ (Figure 4 & Table 2). The signal strength required to engage each of these cytokines was, therefore, essentially the same. No IL-4 or IL-5 was induced at any peptide concentration (data not shown). As far as the co-expression of IL-2 and IFN-γ or of IL-3 and IFN-γ was concerned, the frequency of double positive cells was found to be <8% for the full range of peptide concentration studied (Figure 4). This result reproduced when T cells were tested on days 7, 12, 21 and 55 after the immunization, and applied for dLN, spleen and the CNS. Similar Kf values were obtained when cells isolated from CNS were tested in the presence of the naive syngeneic splenocytes (Table 2) and also the numbers of cytokine co-expressing cells were unaffected by the addition of APC from different sources (LN, spleen, thymus - data not shown). The dissociated production of IL-2, IL-3, and IFN-γ was therefore not a function of the signal strength but seemed to reflect a stable feature of the respective T cells.

**Table 2:** Functional avidity (Kf, mM) of PLP:139-151-specific CD4 T cells producing IL-2, IL-3 and IFN-γ at different stages of chronic-relapsing EAE in SJL mice. ELISPOT assays were preformed at the time points specified with separated CD4 cells (from dLN and Spleen) or unseparated spinal cord cells (the latter were tested with or without added APC, as specified). Legend to Table 1 applies, with the modification that the PLP:139-151-peptide was titrated to establish dose response curves as shown in Figure 4. The Kf values were calculated from the dose response curves as specified in Experimental Procedures*.* The mean and SD of Kf values are shown from three-five independent experiments for each time point.

		Day 7	Day 12	Day 21	Day 55
		No clinical disease	During the first paralysis	During second paralysis	After three relapses of EAE
dLN	IL-2	6 ± 2	5 ± 1	6 ± 3	5 ± 1
	IL-3	5 ± 1	8 ± 2	5 ± 1	6 ± 2
	IFN-g	4 ± 1	7 ± 2	9 ± 3	7 ± 3
Spleen	IL-2	4 ± 2	4 ± 2	5 ± 2	6 ± 2
	IL-3	3 ± 1	6 ± 2	6 ± 3	5 ± 3
	IFN-g	3 ± 1	3 ± 1	5 ± 2	6 ± 2
CNS	IL-2	ND	7 ± 2	8 ± 4	ND
	IL-3	ND	7 ± 2	8 ± 3	ND
	IFN-g	ND	5 ± 2	7 ± 4	ND
CNS plus APC	IL-2	ND	7 ± 2	6 ± 3	ND
	IL-3	ND	7 ± 3	7 ± 2	ND
	IFN-g	ND	4 ± 1	6 ± 2	ND

### 2.6. The Functional Avidity of PLP: 139-151-Specific T Cells is Constant over the Course of EAE

The antigen-specific T cell repertoire is comprised of T cell clones with different avidities for antigen. Studying the CD4 cell response to myelin basic protein (MBP) we found, for example, that T cells that matured in a mouse that did not express MBP were stimulated in average by a 10,000–fold lower MBP dose that T cells that matured in a mouse expressing MBP. The former qualify as high avidity T cells, the latter, as low avidity T cells [41]. Measuring functional avidity gives insights into conceivable repertoire selection processes in the course of EAE, such as the retention of high affinity clones in the CNS or the selective expansion/deletion of this subpopulation in the course of the disease. Moreover, if the different cytokine producing T cells represent different lineages, they might be differently affected by such selection processes. As the data in Table 2 showed remarkable stability of Kf values between anatomic sites, we found no evidence for the retention of high affinity PLP: 139-151-specific cells in the CNS *vs.* the immune periphery (in which case higher peptide doses would be required to stimulate the T cells in the periphery *vs*. the CNS). Also, we found no evidence that over time a selective expansion or deletion of the high affinity PLP: 139-151-specific cells would occur (the unchanged Kf values from day 7 to day 55 in Table 2; if this were the case, then the avidity curves of the T cells would have shifted over time toward a lower or higher peptide range). While repertoire selections have been reported for some T cell responses against foreign antigens [42,43], we found no evidence for them in the autoimmune setting of this EAE model.

### 2.7. Different Distribution of the IL-2, IL-3 and IFN*-γ* -Producing Cells between the Immune Periphery and CNS

**Figure 5:**
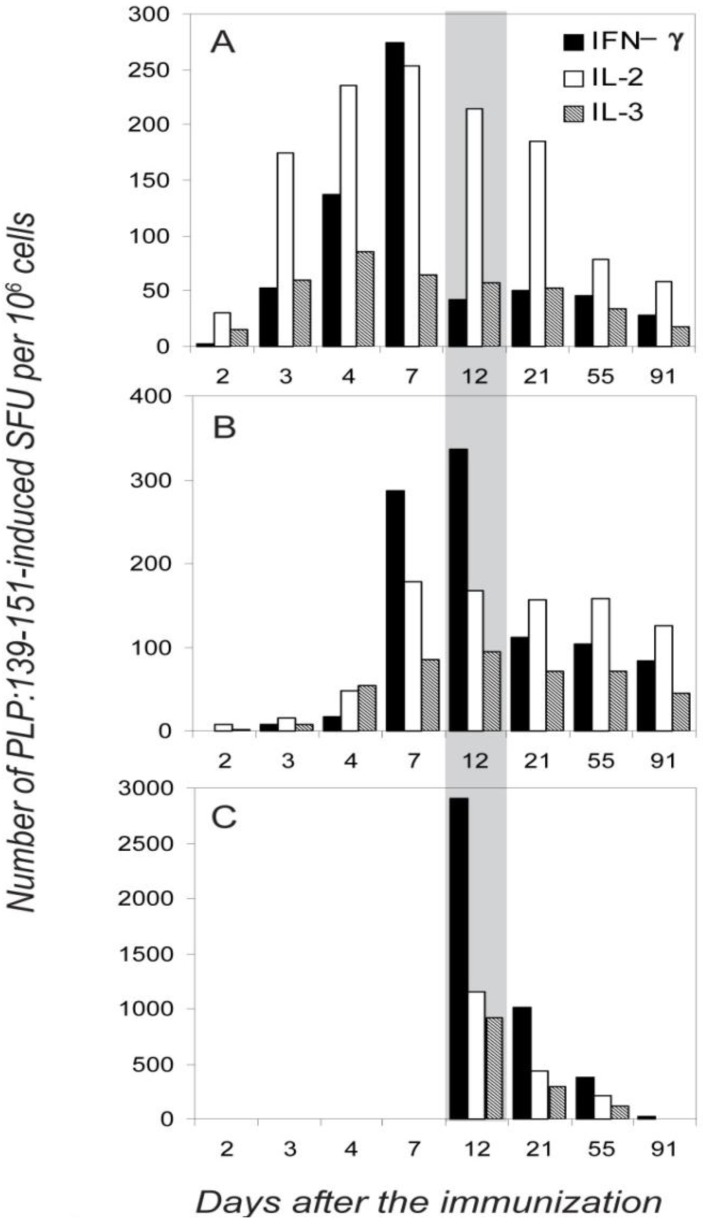
Different organ distribution of PLP:139-151-specific single-cytokine producing T cells during the course of EAE. The frequencies of PLP:139-151-induced IFN-γ, IL-2 and IL-3 spots are shown per one million of mononuclear cells in the dLN (A), spleen (B) and spinal cord (C) measured at different time points after the immunization: days 2, 3, 4 and 7 (before the onset of EAE,) at day 12 (during the first paralysis), and at days 21, 55, and 91 (during the first, second and third relapses of paralytic EAE). The data shown are for T cell activation with 20 mM PLP:139-151. Because the frequencies of double positive cells were consistently <7%, they are not included in the figure. In each experiment mean spot numbers were calculated from three–four replicate wells, SD between replicate wells for all data points did not exceed 10%. For each time point, mean values are shown for data obtained in three independent experiments, testing four mice individually in each experiment. SD between the experiments was <15%. Because statistical analysis included intra- and inter- experiment data and because experimental errors were much lower then differences between data points we aimed to highlight, error bars are not shown.

Figure 5 summarizes the results of five independent experiments in which the frequency of the IFN-γ-and IL-2 (single-cytokine)-producing cells was measured in different anatomic compartments (dLN, spleen and the CNS) over the course of EAE. For each time point, and in each experiment, four mice were tested individually. IFN-γ producing cells appeared first in dLN (where the initial clonal expansion and differentiation occurs after the s.c. immunization), on day 3 where their numbers peaked on day 7 (Figure 5A, and Table 1). With a delay of 2 days, they also appeared in the spleen where their numbers peaked between days 7–12. No IFN-γ producing cells were detected in the CNS until day 12, before the onset of clinical EAE. At the onset of paralysis, ~10 fold higher frequencies were reached in the CNS compared with the peripheral lymphoid tissues. This sudden increase of numbers of IFN-γ producing PLP: 139-151 specific cells in the CNS was accompanied by an instant drop in their frequency in the dLN, but not in the spleen (day 12, Figure 5). Apparently, the IFN-γ producing memory/effector cells generated in the dLN provided the source of cells that migrated to the spleen and the CNS; the cells that migrated to the spleen might have become there temporarily sessile, not participating in the first wave of effector cell migration to the target organ. The more profound subsequent decline in numbers of IFN-γ producing cells in the CNS *vs*. those in the spleen might also argue for this view. 

The frequencies of IL-2 producing cells followed a different pattern. On day 2, IL-2 producers were the first to appear in the dLN, where they were the prevalent cell population throughout the observation period (Figure 5). IL-2 producers appeared with a delay of 2 days in the spleen and subsequently their frequencies did not change in parallel with those CD4 cells producing IFN-γ: the IL-2 producers reached maximal-plateau numbers by day 7 in the spleen, and unlike IFN-γ producers did not undergo rapid frequency changes thereafter. IL-2 producing cells also infiltrated CNS during the onset of the first paralysis, but in contrast to the abundance of the IL-2 producers in the immune periphery, they constituted a minority in the CNS as compared with those secreting IFN-γ. We verified these frequencies by testing the CNS-derived T cells on splenic APC layers, as it was suggested previously that the induction of IL-2 production has more stringent requirements for costimulation than that of IFN-γ [40]. However, as stated before, the frequencies of cytokine producing cells were essentially unaffected by adding APC. The lower frequency of IL-2 spots detected in the CNS, therefore, resulted from the deficit of such cells within the CNS-infiltrate.

The data are consistent with IFN-γ and IL-2 single-cytokine producing cells representing independent subpopulations of memory/effector cells that each follows different migration patterns. The migration pattern of IL-3 producing cells closely followed that of the IL-2 producers (Figure 5).

## 3. Discussion

Our data clearly show that throughout the course of the chronic/relapsing EAE, the vast majority (~95%) of neuroantigen-specific CD4 cells produced either IFN-γ, or IL-2, or IL-3. Neuroantigen-specific CD4 cells that co-expressed these cytokines, or switched between the production of these cytokines, were in the 5% range. In biologic terms, such precision approximates highly regulated processes such as allelic exclusion of TCR chains (4%) [44]. Therefore, the overall type 1 characteristic of the PLP: 139-151-specific T cell response resulted from the joint activity of different CD4 cell subsets that produced either IL-2, or IL-3 or IFN-γ, and not from cytokine secretion by a classic Th1 cell type that would co-express these cytokines.

Single-cytokine producing CD4 cells emerged 2–3 days after the immunization and did not pass through the intermediate stage of differentiation in which they would have co-expressed type-1 and type-2 cytokines. Therefore, multiple division cycles were not required for the acquisition of the single-cytokine producing phenotype. This finding also implies that the IL-2-single producing cell type that we detected do not represent undifferentiated precursor cells for differentiated IFN-γ-producing effectors as was proposed [45].

In contrast to T cell lines and clones that were kept in cell culture for prolonged time periods (involving repeated restimulation with antigen), and that were found to co-express multiple cytokines as a result of prolonged cell cycling [7], in the EAE model we found no evidence for a progression toward a cytokine co-expressing phenotype. It is conceivable that *in vivo* the activated T cells did not pass through the number of activation/division cycles that eventually leads to cytokine co-expression in T cell clones and cell lines. In the course of an immune response, after the initial clonal expansion, the majority of T cells die [46] and evidence has emerged indicating that the T cells most affected are those that underwent more than 4–5 cycles of divisions [47] and those that have reached replicative senescence after >17 cell divisions [8]. It is also possible that the PLP:139-151-specific T cells are not chronically restimulated by the endogenous PLP, but migrate to the CNS, recognize the autoantigen there and die in the target organ without reentering the recirculating T cell pool [48].

The antigen dose and the type of APC could also affect which cytokine is expressed [38,39,40,49]. Accordingly, it was reported for T cell clones, that the expression of IL-2 required a stronger stimulus than IFN-γ [40]. When we titrated the PLP:139-151 peptide over a wide range of concentrations, we did not observe a signal strength-dependent increase in the frequency of cells co-expressing IL-2 or IL-3 with IFN-γ (Figure 4). Additionally, we did not observe IL-4 or IL-5 production at any peptide concentration tested. In fact, the Kf values for the induction of IL-2, IL-3 and IFN-γ producing cells were essentially the same (Figure 4). Using APC from various sources (LN, thymus, spleen) and varying the T cell to APC ratio over a wide range, we did not see an affect on the degree at which IL-2, IL-3, and IFN-γ were co-expressed. All these findings argue against signal-dependent expression of individual cytokines by polyfunctional CD4 memory cells and argue for a stable imprinting to cytokine expression in type 1 memory cells. 

IL-2, IL-3, and IFN-γ - single producing cells also showed similar cytokine-production kinetics (data not shown). These parameters also did not differ for cells obtained from dLN, spleen, or CNS. Cells from all three sites were able to secrete large amount of cytokine a few hours after the restimulation with cognate antigen, suggesting that they do not represent different stages of the differentiation as was suggested previously [45,50]. According to the spot-size distribution analysis, cells isolated from the LN produce higher amounts of IFN-γ as compared to the cells isolated from spleen and CNS (data not shown), therefore, cells residing in the dLN were unlikely to be less differentiated than cells migrating to the periphery. 

The tissue distributions of the single-cytokine-producing T cells that we observed over time (Figure 5) are consistent with the IFN-γ -, IL-2-, and IL-3-producing cells being different lineages that follow different migration patterns. Other interpretations might also be possible, however, to these authors the lineage hypothesis seems the most likely. The IFN-γ-producing cells seem to be generated in the dLN and to migrate from there to the spleen and to the CNS, and only to a lesser extent from the spleen to the CNS. In contrast, IL-2 and IL-3 producers seem to mostly stay in or recirculate within lymphoid tissues (dLN and spleen). IL-3 producers showed similar homing/migration patterns with those of IL-2 producing cells. Therefore impaired ability of IL-2 producers to migrate to the CNS is not unique and cannot be explained only by the central memory/effector memory dichotomy. In spite of their lower relative frequencies in the periphery as compared to IFN-γ producers, IL-2 producing cells still were abandoned in the CNS, suggesting that at least a substantial fraction of them lost LN homing receptors and migrated to the periphery. The absolute frequencies of all single-cytokine expressing cell subpopulations were declining in parallel in the draining LN and in the CNS during the progression of chronic-relapsing EAE, suggesting that one subpopulation does not develop from the others (IL-2-producers being precursors for IFN-γ for instance). These single-cytokine producing subpopulations were formed 2–3 days after the immunization and were stable during a 3 month observation period during which mice had three-four episodes of chronic-relapsing EAE. There was no avidity- or cytokine profile maturation during chronic exposure to self antigen.

## 4. Experimental Section

### 4.1. Animals and Immunizations

SJL/J mice were purchased from The Jackson Laboratories and bred at CWRU under specific-pathogen-free conditions. Female mice were immunized at 6–8 wk of age. PLP peptide 139–151 [32] was purchased from Princeton Biomolecules (Columbus, OH, USA). IFA was purchased from Gibco BRL, (Grand Island, NY, USA) and CFA was prepared by mixing inactivated *M. tuberculosis* H37RA (Difco Laboratories, Detroit, MI, USA) at 1 mg/mL into IFA. PLP:139-151 peptide (2 mg/mL) and PBS were mixed 1:1 with adjuvant and was injected once in 100 μL (100 μg/animal), subcutaneously (s.c.). Pertussis toxin (0.2 μg) was injected twice, once immediately and once 24 h after immunization [32,51]. 

### 4.2. Cell Preparations

Cells from the CNS were prepared as follows. After sacrificing, the animals were perfused with PBS, the spinal cords were removed from the entire vertebral column and placed into DMEM medium. The spinal cord was disrupted with the back of a syringe. The resulting cell suspension was filtered through a Falcon Cell Strainer 2350 (Becton Dickinson, San Jose, CA, USA). The cells were washed twice with DMEM and subsequently counted. As we found in our previous work, infiltrating mononuclear cells isolated from spinal cords of mice with EAE contained 19%–43% CD4 cells. We also demonstrated that for functional T cell assays, there is no need for further ficoll/percoll purification of mononuclear cells, and for addition of APC [52,53]. Single cell suspensions from draining and non-draining LN and the spleen were prepared as previously described [6]. CD4 cell fractions were obtained with >97% purity by passing single cell suspensions over ‘Mouse CD4 Subset Columns’ (R & D Systems, Minneapolis, MN, USA) according to the manufacturer’s instructions.

### 4.3. Two-Color Cytokine ELISPOT Assays

Plates (MSIPN4W, Millipore, Billerica, MA) were coated overnight at 4 °C with the cytokine-specific capture antibodies specified below. The plates were then blocked with 1% BSA in PBS for 1 h at room temperature and washed 4× with PBS. Subsequently, irradiated (3,000 rad) LN or splenic APC from naive, syngeneic mice were added (5 × 10^5^ cells/well, or as specified). Freshly isolated CD4 or single-cell suspensions from spinal cords were plated in serial dilution in 2–4 replicate wells with or without the nominal antigen or control antigens. The assay medium was serum-free HL-1 (Biowhittaker, Walkersville, MD.) supplemented with 1 mM L-glutamine. After 48 h of cell culture in the incubator at 37 °C, the cells were removed by washing 3 × with PBS and then 4 × with PBS containing 0.05% Tween-20 (PBST). The two detection antibodies were added simultaneously and the plates were incubated at 4 °C overnight, after which they were washed 3 × with PBST. For the biotinylated detection mAbs, the streptavidin-ALPH (alkaline phosphatase) conjugate (Dako Corp., Carpenteria, CA, USA) was added (at 1:2000 dilution), incubated for 2 h at room temperature and removed by washing twice with PBST and twice with PBS. The Vector Blue ALPH substrate (Vector Laboratories, Burlingame, CA, USA) was added for 15–30 min. Then, after washing twice with PBS, the AEC substrate (Pierce, Rockford, IL, USA) was added and left for 20–40 min. The following coating mAbs were used for IL-2, IL-3, IL-4, IL-5, and IFN-γ: JES6-1A12 (5 μg/mL), MP2-8F8 (5 μg/mL), BVD4-1D11 (2 μg/mL), TRFK5 (5 μg/mL), and R46A2 (2.5 μg/mL). The combinations of detection antibodies for the IL-2:IFN-γ, IL-3:IFN-γ, IL-4:IL-5, IFN-γ:IL-5, IL-4:IFN-γ, and IL-2:IL-5 assays were: JES6-5H4-biotin:XMG1.2-HRP, MP2-43D11-biotin:XMG1.2-HRP, BVD4-24G2-biotin: TRFK4-HRP, XMG1.2-biotin:TRFK4-HRP, BVD4-24G2-biotin:XMG1.2-HRP, and JES6-5H4-biotin:TRFK4-HRP (all antibodies were from Pharmingen, San Diego, CA). Horse radish peroxidase (HRP)-labeling of antibodies was performed by the periodate method [54]. The combination of JES6-5H4-biotin or MP2-43D11-biotin detection mAbs with unlabeled affinity-purified goat polyclonal antibodies to mouse IFN-γ (R&D Systems, Minneapolis, MN) followed by the mixture of streptavidin-ALPH with HRP-labeled rabbit-anti-goat antibodies (Zymed, San Francisco, CA ) were also used for IL-2:IFN-γ, IL-3:IFN-γ two-color ELISPOT. This variant gives the same sensitivity for the detection of single and double-cytokine producing cells, and permits use of off-the-shelf reagents if HRP labeling cannot be performed. The detection antibody concentrations were as follows: JES6-5H4-biotin (2 μg/mL), MP2-43D11-biotin (2 μg/mL), BVD4-24G2-biotin (2.5 μg/mL), TRFK4-HRP (2 μg/mL), XMG1.2-HRP (2 μg/mL), XMG1.2-biotin (2 μg/mL), polyclonal goat-anti-mouse IFN-γ (0.5 μg/mL). Computer-Assisted Two-Color ELISPOT Image Analysis was performed as previously described [6] using a Series 6 ImmunoSpot Image Analyzer (CTL Analyzers LLC, Cleveland OH). 

### 4.4. Measurements of Functional Avidity

T cell cytokine dose-response curves obtained by ELISPOT are closely approximated by a sigmoidal function in the Log scale, or a hyperbolic function on a linear peptide scale, asymptotically reaching a plateau at high concentrations of the peptide (see Figure 4). Based on the similarities between receptor-ligand binding isotherms and dose response curves of T cell function, functional avidity (Kf) was defined as the concentration of peptide that leads to the activation of 50% maximal number of T cells [55]. As defined, functional avidity represents the integrated characteristic of the entire heterogeneous T cell population. Kf values were calculated from the ELISPOT dose-response curves in Log scale by approximation with sigmoidal function: *Y = a/(1 + exp(-(X + b)/c))*, where *‘X’* is a log_10_ of the recall peptide dose and *‘Y’* is the number of spots detected at each peptide concentration. Furthermore*, ‘a’*,*’b’*, and *‘c’* are the parameters of non-linear regression, *‘b’* being equal to the Lg (Kf) and *‘a’* being the asymptotic limit for the maximal number of cells capable of producing cytokines in the T cell population tested.

## 5. Conclusions

It is tempting to postulate that the commitment of the memory CD4 cell to express a particular cytokine gene upon re-encountering antigen is the result of instructed differentiation, similar to the well-defined differentiation events that lead to the generation of memory cells that produce either IFN-γ or IL-4, or IL-17. Evidence has emerged that the differentiation into IL-5-producing memory cells has different requirements than the maturation of IL-4-producing memory cells [56,57,58]. Therefore, the delegation of individual cytokines to distinct CD4 cell lineages might be a fundamental principle that assures precise delivery of cytokine-mediated effector functions. 

A recent study performed on human T cells provided confirmatory results to those reported here [2]. Individual T cells were distributed into microwells that accommodated only one cell each, and cytokines were measured in the supernatants of these individual wells/cells. It was shown that each cell produced only one cytokine, and that apparent double cytokine production resulted form cytokine switching – but even the double producers produced at a given time point only one cytokine. 

**Figure 6:**
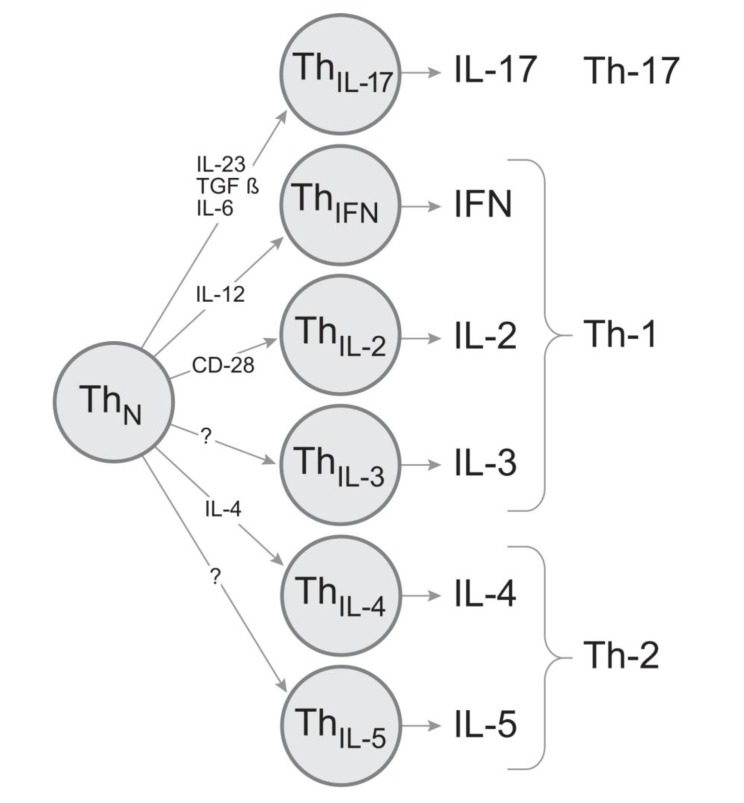
Proposed model for CD4 cell cytokine sublineages. The production of each key effector cytokine is delegated to a separate lineage. They arise through instructive differentiation driven by specific cytokine environments and/or signals. The individual cytokine lineages are clonally expandable permitting to deliver precise effector functions.

High precision in the engagement of cytokine- mediated effector functions should be a prerequisite for successful CD4 cell-mediated host defense. By operating with only few sets of cytokines, as the classic Th1/Th2/Th17 models predict, the repertoire of biologic messages conveyed would be limited to few stereotypic response types. Stochastic cytokine expression patterns in T cells, in the absence of strict subsequent selection, also cannot result in predefined and precise CD4 cell effector functions. There might, therefore, have been considerable evolutionary pressure for independent, highly regulated cytokine expression by CD4 cells. This would be best accomplished with individual CD4 cell sublineages that express a single cytokine (or certain permitted pairs of combinations of cytokines). By subjecting the engagement and the clonal expansion of these lineages to stringent control during the primary immune response, a wide variety of highly defined effector functions can be assured (Figure 6). According to this model, it should be possible to selectively induce or inactivate the individual cytokine-producing subpopulations and by so doing promote the desired and suppress the harmful T cell effector functions for the immune therapy of T cell-mediated autoimmune diseases.
